# Proceedings of the Philadelphia County Medical Society

**Published:** 1877-01-20

**Authors:** Benjamin Lee

**Affiliations:** The Vice-President, in the Chair


### PROCEEDINGS OF THE PHILADELPHIA COUNTY MEDICAL SOCIETY.

THE VICE-PRESIDENT, DR. BENJAMIN LEE, in the Chair.

At a conversational meeting, held Arpil 26, 1876, after the reading of Dr. Taylor's paper, for which a vote of thanks was tendered to the author, the subject of the "Influence of Maternal Impressions" was discussed.

Dr. Wm. Goodell thought that there is more truth in the popular belief than physicians are willing to concede. He had noticed, however, of late, a growing disposition on the part of scientific men to treat the subject with more consideration. As a matter of fact, the spermatozoon, a microscopic cell, is capable of transmitting and reproducing paternal traits, both mental and physical. Hence it would seem highly probable that the mother, who contributes so much to the structure of the new being, would be more likely to impress upon it her dominant traits during its plastic stage, and that unusual excitement or agitation on her part during this period would be exceedingly liable to disturb the development of the fœtus. The following case is one in point: He was called to attend a lady, whose husband (a physician) was possessed with the idea that the child would be born with some deformity of the genital organs. This, indeed, Dr. C. found to be the case, for at the birth the foreskin was entirely wanting, and the glans encircled by a ring of granulations, presenting the appearance of a recent circumcision. This occurrence was attributed to the fact that the mother, during early pregnancy, had taken extraordinary interest in a description of this Hebrew rite, which her husband had been invited to witness in a neighbor's child. Among the Jews, children are not infrequently born circumcised; and this curious fact is not to be explained by the law of heredity alone.

Dr. J. S. Eshleman said that there was undoubtedly a germ of truth in the popular belief, but he always endeavored to explain away such morbid impressions, when they existed, for the sake of the mother, whose health might be affected by dwelling on them.

The following case in Dr. E.'s practice appeared at first to strongly confirm the opinion that maternal impressions may affect the development of the fœtus: An Irish woman was firmly convinced that her child would be born with a tumor "on its seat," because some one had described such a congenital growth in her presence. At the birth a large prominence was discovered in the child's perineum to confirm her impression. This tumor was subsequently removed by Prof. Gross, who pronounced it a fœtal cyst, due to a germ that had been blighted in its development. If the nature of the growth had not been demonstrated, this case would have been confirmation strong; but it would be difficult to see how a mental impression on the mother could be powerful enough to introduce into the uterine cavity the germ of another child.

Dr. John G. Stetler said that it was an acknowledged physiological law that the first pregnancy influenced subsequent conceptions, but thought that it acted locally through the impression on the uterine tissues probably, rather than by means of the nervous system.—*Med. Times.*

